# Expectations: How and when do they contribute to placebo analgesia?

**DOI:** 10.3389/fpsyt.2022.817179

**Published:** 2022-09-06

**Authors:** Sophie Rosenkjær, Sigrid Juhl Lunde, Irving Kirsch, Lene Vase

**Affiliations:** ^1^Department of Psychology and Behavioural Sciences, School of Business and Social Sciences, Aarhus University, Aarhus, Denmark; ^2^Program in Placebo Studies, Beth Israel Deaconess Medical Center Harvard Medical School, Boston, MA, United States

**Keywords:** placebo effects, expectations, prediction, placebo analgesia, hope

## Abstract

In placebo research, expectations are highlighted as one of the most influential subjective factors. While some studies have shown a relationship between expectations and pain relief, others have not. However, little is known about how methods of assessment of expectations may affect these conclusions. One of the fundamental considerations is that participants in placebo trials rate their expectations when prompted to rate them on scales in advance, but are less likely to report their prior expectations, when asked to report their experience retroactively in an unprompted manner, often expressing, for example, prior hope or wishes of recovery. This article presents previously unpublished data to elucidate and explore the concepts highlighted by individuals in a placebo analgesia trial when assessed in a prompted and unprompted manner. The data corroborates the role of expectations involved in placebo effects, particularly in placebo analgesia. Thus, the question may be a matter of *how* and *when* expectations contribute to placebo effects, rather than *if*.

## Introduction

In placebo research, expectations have long been emphasized as crucial to the shaping of placebo effects (1–4). Several studies have shown that participants’ expectations significantly contribute to placebo effects (5–7), while other studies have not found this relationship (8, 9). This discrepancy may, among other things, be a result of differences in the way expectations are assessed. When examined in studies, expectations are rarely defined, and no common definition exists. Therefore, expectations assessed in placebo research may reflect various constructs or different aspects of the same construct. In addition to the need for a common definition of expectations, there is a need for awareness of the way we tap into expectations, and this latter point is the subject of the present article. Theories of expectations in placebo effects have rightfully been criticized as needing to be more nuanced (10). Previous literature has made efforts to elaborate on the theory of expectations in placebo effects (3, 10, 11). In the first part of the present article, selected aspects of expectancy theory of relevance for assessing expectations are briefly highlighted, and in the second part, this theory is illustrated and corroborated with examples of how expectations have been assessed in a placebo study with both prompted and unprompted data. The prompted data has previously been published (12). The unprompted data was collected in the same study, but not previously published. However, since the first publication, more debate has arisen about the role of expectations in placebo studies (8, 10, 13), which makes the data relevant to look further into. The prompted and unprompted data yield different results within the same study and can therefore contribute to nuance the relation between expectations and placebo analgesia. The unprompted data is used in an exploratory and hypothesis-generating manner to add to the debate about how to assess and evaluate the role of expectations in placebo effects. The present article presents a short overview of the pressing issues which we believe one should be aware of when including expectation assessments in placebo studies. Finding the solutions for these issues and providing conclusive definitions are beyond the scope of this article and would be relevant to consider in joint efforts or future expert consensus.

## Selected aspects of expectancy theory

This section presents selected aspects of expectancy theory that contribute to important distinctions in the assessment of expectations, but the list is by no means exhaustive.

To make a broad overview and distinction, we use Laferton and colleagues’ critical review of expectation concepts in medical treatment (14), which synthesizes relevant elements in understanding expectations. The review distinguishes between (1) expectations as future-directed beliefs focusing on specific events or experiences which may or may not happen and (2) concepts referring to what patients would like to happen (i.e., hopes or desires), which have also been termed ideal expectations or fantasies (14). Furthermore, the model of expectations by Laferton et al. states that patients have so-called timeline expectations as to the temporal aspect of behavior, treatment, disease, and outcomes (14). Such a temporal dimension to subjective expectations may be similarly relevant in placebo studies when participants receive information or have expectations about *when* to expect benefits from treatment to emerge or subside.

### Probability and emotion

Previously, expectations and hope have been conceptualized as both overlapping and separate phenomena (15, 16). For example, in interviews of participants’ experience of participating in a study, some studies report expectations which overlap with hope (17), while others have found that hope is more prevalent (16) and have suggested that hope may be dominant in patients with chronic pain compared to healthy participants (18). Hope, like expectations, has no consistent definition, but it is generally agreed that hope refers to desirable future events or experiences (15, 19). Open label placebo trials, wherein placebo treatment is given openly, and participants are informed that they are receiving an inert treatment, illustrate that expectations have a complex interaction with hope. In open label trials, few participants may believe that they can expect symptom reduction directly whereas many participants are simply hopeful or even skeptical toward symptom relief (10, 20). In this way, the role of hope and expectations in open label trials may differentiate from other placebo trials. Even so, open label placebo trials have been successful in inducing placebo effects, despite participants being aware that they are receiving inert treatment (20–22).

### Levels of consciousness

Commonly, placebo effects have been modulated through expectations (1) assessed by verbal ratings of expectations which are consciously available (23). However, placebo effects have also been induced through conditioning or even subliminal procedures, without conscious awareness of these subliminal stimuli (24–26). Therefore, it seems that placebo effects may not always involve conscious expectations, but may possibly be induced through other, not consciously available, predictive processes in the brain (9, 27). It has been discussed how subliminally induced placebo effects interplay with conscious expectations (18). It is still unknown whether subliminal cues lead to changes in conscious expectations of pain, even if patients are not aware of these cues (28). Even so, subliminal aspects of placebo effects may be important in further nuancing theories of expectations. Future research in subliminally induced placebo effects may help uncover various paths to induce placebo effects and the extent to which conscious expectations are needed in the shaping of placebo effects.

### Temporal features

In addition to the level of consciousness of expectations, recent studies suggest that it may be crucial *when* expectations are assessed (29, 30). New lines of research on placebo effects have begun to focus on the temporal aspects of placebo effects and expectations. Exemplifying this, studies on healthy participants have shown that external time cues, i.e., information on *when* a treatment is expected to take effect, influence the onset and time course of placebo effects (29, 30). Furthermore, focusing on the participants’ ratings of expectations *throughout* their study participation, expectations of pain relief have been found to significantly predict perceived pain levels at different time points–even when controlling for a gradual learning effect across test days (31). In other words, these findings point to a substantial contribution from the participants’ expectations for pain relief that exceeded their prior experiences obtained throughout the study.

These theoretical stances point to expectations as a complex concept, which is not fully assessed through unidimensional measures. To further investigate this, we consider data from the study explained below, which tapped into expectations through two different angles: prompted and unprompted measures.

## Examples of prompted and unprompted expectation assessment in placebo studies

### Prompted expectation assessment

**FIGURE 1:**
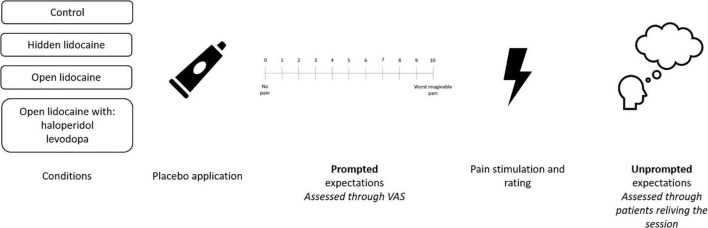
Order of procedures and expectation approaches in the study of placebo effects in chronic neuropathic pain patients (12).

Expectations are dependent on the method through which they are assessed. Exemplifying different approaches, prompted measures refer to measures explicitly asking about expectations, while unprompted measures do not specifically inquire about expectations. The importance of distinguishing between these types of assessments is illustrated by data from a study investigating placebo interventions in 19 patients suffering from chronic neuropathic pain (12). One of the co-authors of the present article (L.V.) supervised the study and the study design is presented in Figure 1. In the study, patients went through open and hidden applications of lidocaine and a no treatment condition. After application of lidocaine and before assessment of ongoing neuropathic and evoked (pin-prick evoked/windup-like) pain, expectations were assessed using a visual analogue scale (VAS). When asked in a prompted manner using the VAS, all participants gave an indication of their expectations. These expectations accounted for 41.2% of the variance in ongoing and evoked neuropathic pain (12). Thus, prompted expectations were found to significantly predict pain.

### Unprompted expectation

**FIGURE 2:**
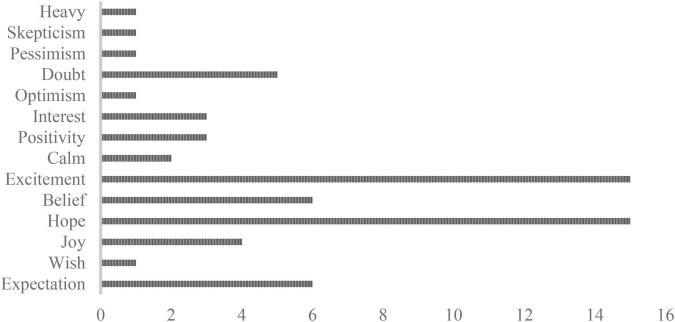
Number of times experiences were expressed by the chronic neuropathic pain patients across the open conditions (open, open + haloperidol, and open + levodopa) of the study (12). Sixty-four experiences were reported across the three conditions which were distributed as presented in the figure.

The same 19 chronic neuropathic pain patients also underwent an inquiry of their experiences in which they were not directly asked to report what their expectations had been. This data has not previously been published and is presented here to illustrate and debate how differences in the way expectations are assessed may influence findings. At the end of each treatment session, patients were asked to relive the session and describe their experiences freely with regard to their positive and negative experiences. Participants expressed their experience through a single sentence, for example “I very much hoped that the treatment would work.” Experiences from the three open conditions (open lidocaine, open lidocaine with haloperidol, open lidocaine with levodopa) were synthesized and analyzed using thematic analysis, regarding the word that best described what was expressed by each participant, and themes are displayed in Figure 2. The patients indicated their experiences in each condition. A total of 56 positive and eight negative experiences were expressed across the three conditions. A range of experiences including elements of expectations were reported, as illustrated in Figure 2. Yet, in this free report expectations were only directly expressed six times. Thus, using this unprompted approach, expectations do not seem crucial for the experience and prediction of pain.

This study clearly illustrates that the way expectations are assessed impacts the resulting conclusion about their role. While the prompted measurement reflects the typical assessment of expectation in placebo trials (32–34), the unprompted data may tap into the other aspects of expectations which are related to expectations or play a role in placebo analgesia, e.g., hope (16). There are clear advantages and limitations of both types of assessments: Prompted measures directly target expectations and, in the case of VAS scales, are easily relatable to pain measures. This prompting may also be a limitation, as the prompting may direct the answer or be too narrowly focused on a single dimension. Furthermore, variability in definitions of prompted measures could impact the results. For example, it has previously been shown that there are different and even opposing effects in deceptive and double-blind placebo groups, when addressing the subjective likelihood (e.g., “How likely is it that your pain will be reduced after treatment?”) and the expected magnitude (e.g., “What do you expect your pain to be after treatment?”) (35). Thus, the framing of prompted measures may lead to important variability.

The unprompted data, however, allows for further nuancing of the dimensions of expectation which can contribute to placebo effects and does not predispose a particular answer. Limitations of unprompted data make it more complex to quantify and relate to other (prompted) measures.

Importantly, the prompted expectations were assessed before the pain experience, while the unprompted expectations were assessed retrospectively, which could impact the findings. It has been shown that pain ratings change when addressed retrospectively, though maintaining an association with expectations both in concurrent and remembered ratings (4). Similarly, expectation ratings may change when addressed retrospectively. In interviews on patient experiences, Kaptchuk and colleagues (16) showed an important role of retrospection and highlighted that memory bias may shape the subjective experience of treatment outcomes. Thus, memory bias could result in the differences seen between the prompted and unprompted data, rather than inherent differences in the aspects of expectations which are targeted by each measure. In the study by Kaptchuk and colleagues (16), interviews were conducted over six weeks concurrently with ongoing placebo treatment. In contrast, the unprompted expectations of the present article were assessed immediately after completion of the test session. Thus, the time points of assessment in the two studies differed notably. Still, both studies found that expectations may be less prevalent when addressed in a more open fashion. This suggests that prompted and unprompted measures of expectations may lead to different findings independently of whether they are measured concurrently or retrospectively. Yet, studies that prospectively measure expectations in prompted and unprompted manners are needed to tease time and measurement apart.

That expectations seem to be more prevalent in prompted measurements compared to unprompted measurements could also reflect the involvement different levels of consciousness. In this way, consideration of unprompted measures could be valuable to nuance the theory of expectations and understand the experience of placebo analgesia.

The study presented in this article further underlines how different ways of tapping into expectations may not reflect the same construct or even reflect actual assessments of expectations. That is, some ways of tapping into expectations may be central to measuring them, while others may not adequately measure or reflect the expectations of participants in placebo studies. The study shows that this can result in the conclusion that expectations are important when looking at the prompted rating *or* not important when looking at the unprompted assessment in placebo analgesia effects.

### Probability, emotion, and temporal aspects

Results from the study of chronic neuropathic pain patients also illustrate important dimensions of expectations and experiences in placebo studies, corroborating the highlighted theory elements from Laferton et al. (14). The broad range of experiences portrayed in Figure 2 can be divided into two categories: Whereas one is future-directed with clear relations to future events or experiences (e.g., belief, hope, wish, and expectation), the other is not related to a future outcome (e.g., calm, joy, and interest). The category of future-related concepts includes expectations and other concepts which may coexist or overlap with expectations. Thus, rather than developing several separate theories about each future-directed subjective experience and their influence on placebo effects, expectations may further be subdivided into probability-related or emotion-related. We suggest that this division would result in expectation and belief on the one hand (probability-related) and hope and wish on the other hand (emotion-related). The exact elements of this division may, of course, change depending on context. Ideally, both aspects of expectations should be considered and assessed in placebo studies to further develop the theory–along with continuously tapping into expectations at different time points throughout these studies to capture their temporal development, persistence, and/or change over time. In addition, it is important to be aware that the way expectations are induced may have a significant impact on how they manifest in assessments. Open label placebo, conditioning procedures, or verbal suggestions may not manifest and be assessed in the same manner, and potentially different assessments should be used to fully capture the broad spectrum of expectations.

### Temporal features

The abovementioned study of neuropathic pain patients did not directly investigate temporal aspects of expectations. However, in another study of chronic pain patients (17) involving prompted and unprompted expectation measures, it was shown that these measures may also differ regarding the temporal development. While the prompted measurement of expectation showed a change over time (expectations to pain relief on a VAS were higher over time), the unprompted measurement showed that once expectations were established, patients’ focus of attention appear to change away from their expectations (17). Thus, it is possible that there may be differences in the way prompted and unprompted measures develop over time and potentially the way they tap into expectations, but this needs to be investigated systematically in future studies.

## The future of expectation assessments in placebo studies

Placebo studies increasingly include assessments of expectations. This inclusion offers the possibility of comparing expectations across different study settings and medical conditions. However, this also highlights the need for clarifying which concepts or which aspects of expectations we are dealing with.

As illustrated above, the question of whether expectations contribute to placebo effects is complex and appears to be dependent on how, when and under which conditions, they are assessed, as different approaches seem to lead to different results and conclusions. Thus, in future studies, it will–as a minimum–be important to pay attention to the extent to which expectations involve different dimensions of future-directed experiences. These may be probability-related or emotion-related, may be present at different levels of consciousness, and may include temporal aspects. All of these aspects should be kept in mind in future studies when approaching expectations both in a prompted and unprompted manner. The measurement of expectations in placebo studies demands more attention. Inclusion of more detailed expectation assessments in studies, could further lead to comparison of which aspects of expectations are important in certain contexts, that is the *when* and *where* of expectations.

## Data availability statement

The original contributions presented in this study are included in the article/supplementary material, further inquiries can be directed to the corresponding author.

## Author contributions

SR and SL drafted and revised the manuscript. LV provided the data and critically revised the manuscript. IK critically revised the manuscript. All authors contributed to the article and approved the submitted version.
